# Complete Genome Sequence of Escherichia coli Myophage Mansfield

**DOI:** 10.1128/MRA.01038-19

**Published:** 2019-09-19

**Authors:** Genevieve M. D’Souza, Kathryn Klotz, Russell Moreland, Mei Liu, Jolene Ramsey

**Affiliations:** a Center for Phage Technology, Texas A&M University, College Station, Texas, USA; b Department of Animal Science, Texas A&M University, College Station, Texas, USA; Queens College

## Abstract

Mansfield is a PB1-like Escherichia bacteriophage with a 68,120-bp genome and a predicted 3,673-bp direct terminal repeat. This myophage encodes 105 proteins, for which 32 functions were predicted.

## ANNOUNCEMENT

Escherichia coli is a commensal Gram-negative bacterium that thrives in the intestinal section of the gastrointestinal tract (1), but some strains are pathogenic (2). E. coli O157:H7 is one of the most virulent serotypes, with its strains inducing severe bloody diarrhea and dehydration in infected patients (3). Although humans are a primary host for symbiotic E. coli strains, livestock have also been identified as carriers and shedders of strains that are commensal and pathogenic to humans (4). With the rise of antibiotic resistance, bacteriophages are being considered as a precision alternative medicine for eliminating E. coli specifically (5). Here, we present the genome sequence of myophage Mansfield, which infects E. coli.

Mansfield was isolated from filtered (0.2-μm pore size) stream water in College Station, TX, by propagation on its host, E. coli 4s, grown in Luria broth/agar aerobically at 37°C via the soft agar overlay method (6, 7). Mansfield genomic DNA was purified using the Promega Wizard DNA clean-up system described by Summer (8). A library prepared with a TruSeq Nano low-throughput kit was sequenced on an Illumina MiSeq platform using paired-end 250-bp reads with V2 500-cycle chemistry. The 414,121 sequence reads from the index containing the phage genome were quality controlled using FastQC (http://www.bioinformatics.babraham.ac.uk/projects/fastqc/), and a complete genome was assembled via SPAdes v3.5.0 (9), with 297.7-fold coverage after trimming with the FASTX-Toolkit 0.0.14 (http://hannonlab.cshl.edu/fastx_toolkit/). PCR (forward primer, 5′-CGACACATTCGGTCCACTAA-3′; reverse primer, 5′-TATTGAGCGTTCCCTCGAAAG-3′) and Sanger sequencing were used to close the genome. Gene calling was completed with Glimmer v3.0 and MetaGeneAnnotator v1.0 (10, 11). Gene functions were predicted using InterProScan v5.33-72, TMHMM v2.0, and BLAST v2.2.31 at their default settings, cross-referencing hits for BLAST at a 0.001 maximum expected value cutoff versus the NCBI nonredundant, UniProtKB Swiss-Prot, and TrEMBL databases (12–15). Additional evidence came from the HHSuite v3.0 tool HHpred (multiple-sequence alignment [MSA] generation with HHBlits ummiclus30_2018_08 database and modeling with PDB_mmCIF70) (16). TransTermHP v2.09 was used to annotate Rho-independent termination sites (17). The absence of tRNA genes was determined using ARAGORN v2.36 (18). Genome sequence similarities were calculated by progressiveMauve 2.4.0 (19). All tools are hosted in the Center for Phage Technology Galaxy instance, and annotation was performed in Web Apollo (https://cpt.tamu.edu/galaxy-pub) (20, 21). To determine Mansfield’s morphology, samples were negatively stained with 2% (wt/vol) uranyl acetate and viewed by transmission electron microscopy at the Texas A&M Microscopy and Imaging Center (22).

The Mansfield genome is 68,120 bp, with a G+C content of 46.14%. The 105 protein-coding genes are at a 93% coding density, and 32 have a predicted function. Mansfield’s genome was opened at the boundary of 3,673-bp direct terminal repeats predicted by PhageTerm (23).

The two phages most closely related to Mansfield are PB1-like phages of the pbunaviruses, *Escherichia* phage ECML-117 (GenBank accession number JX128258) and *Escherichia* phage FEC19 (GenBank accession number MH816966), having 90.89% nucleotide similarity and 90 proteins in common with phage ECML-117, and 90.52% nucleotide similarity with 89 proteins in common for phage FEC19 (24, 25).

### Data availability.

The genome sequence and associated data for phage Mansfield were deposited under GenBank accession number MK903282, BioProject accession number PRJNA222858, SRA accession number SRR8893603, and BioSample accession number SAMN11414488.
